# Prevalence of fatigue in patients 3 months after stroke and association with early motor activity: a prospective study comparing stroke patients with a matched general population cohort

**DOI:** 10.1186/s12883-015-0438-6

**Published:** 2015-10-06

**Authors:** Thorlene Egerton, Anne Hokstad, Torunn Askim, Julie Bernhardt, Bent Indredavik

**Affiliations:** Department of Neuroscience, Faculty of Medicine, Norwegian University of Science & Technology, Trondheim, Norway; The Stroke Unit, Department of Medicine, St Olavs Hospital, University Hospital of Trondheim, Trondheim, Norway; Department of Physiotherapy, Faculty of Health Education and Social Work, Sør-Trøndelag University College, Trondheim, Norway; Florey Institute of Neuroscience and Mental Health, Melbourne, Australia

**Keywords:** Stroke, Fatigue, Physical activity, Early mobilization, HUNT

## Abstract

**Background:**

Fatigue is a common complaint after stroke. Reasons for higher prevalence are still unclear. This study aimed to determine if fatigue prevalence in stroke patients is different to that of age and gender matched general population controls, and to explore whether early motor activity was associated with reduced likelihood of fatigue three months after stroke.

**Methods:**

This was a prospective multicenter cohort study of stroke patients admitted to eleven regional Norwegian hospitals, within 14 days after stroke. Stroke patients (*n* = 257) were age and gender matched to participants in a general population health survey (HUNT3-survey) carried out in a regional county of central Norway. The single-item fatigue questionnaire from the HUNT3-survey was administered to both groups to compare prevalence. The association between early motor activity (*time in bed, time sitting out of bed*, and *time upright)* and fatigue at three months after stroke (Fatigue Severity Scale) was tested with logistic regression. Simple models including each activity outcome, with adjustment for stroke severity and pre-stroke function, were tested, as well as a comprehensive model that included additional independent variables of depression, pain, pre-stroke fatigue, age and gender.

**Results:**

Prevalence was higher after stroke compared with the general population: 31.1 % versus 10.9 %. In the simple regression models, none of the early motor activity categories were associated with fatigue three months after stroke. In the comprehensive model, depression, pain and pre-stroke fatigue were significantly associated with post-stroke fatigue. Time in bed through the daytime during hospital stay approached statistical significance (*p* = 0.058) with an odds ratio for experiencing fatigue of 1.02 (95 % CI 1.00-1.04) for each additional 5.4 minutes in bed.

**Conclusions:**

Stroke patients had higher prevalence of fatigue three months after stroke than the age and gender matched general population sample, which may be partly explained by the stroke population being in poorer health overall. The relationship between early motor activity (and inactivity) and fatigue remains unclear. Further research, which may help drive development of new treatments to target this challenging condition, is needed.

## Background

Fatigue is described as a “constant weariness unrelated to previous exertion levels and not usually ameliorated by rest” [1]. Perceptions of fatigue are a common complaint among older people and for those with a range of chronic diseases including stroke. The prevalence of fatigue in the general population has been variably reported from 5 to 47 %, depending on the population studied, the questionnaire used, and the threshold score used to differentiate those with fatigue from those without [2–5]. Prevalence appears to increase with the number of chronic diseases [6, 7], and is higher in women [8], but findings are inconsistent for age [3, 9].

Prevalence is elevated even further following stroke, ranging from 35 to 92 % [10], again depending on the tool used to measure fatigue, but also depending on the time since stroke and sample selection strategies [10–13]. Post-stroke fatigue (PSF) is distressing and debilitating. It is associated with higher levels of dependency [14, 15] and poorer quality of life [16]. It also independently predicts institutionalization and mortality after stroke [17, 18]. Fatigue is rarely assessed in clinical practice and poorly managed, largely because strong evidence supporting effectiveness of fatigue-reducing interventions, either for fatigue in general, or for fatigue unique to stroke patients, is lacking.

Fatigue after stroke is complex, and while fatigue can be experienced secondary to medications, sleep disorders and/or medical complications [19], it is probable that PSF also relates to the brain injury itself [14, 17, 20–22]. Ongoing fatigue may be compounded by reduced activity and subsequent deconditioning, particularly in the sub-acute phase, perhaps in combination with the increased energy cost of movement due to impairment [22–25]. Our current understanding of the biology of fatigue is limited. Understanding how PSF may differ from other fatigue is clinically important as unique management options may be required. If deconditioning and movement inefficiency play a crucial role in the experience of fatigue later after stroke, increased physical activity opportunities and movement training may be further endorsed as a treatment approach.

Several previous studies have investigated risk factors for PSF. Evidence suggests the main predictors for fatigue in the sub-acute phase are depression [11, 26], pre-stroke fatigue [26–28], and pain [29, 30]. Recent evidence suggests that activity early after stroke (step count at one month) predicts fatigue later after stroke (six and 12 months) [31]. However, knowledge is limited on the role of physical activity on fatigue levels for stroke patients, especially in the early phase after stroke.

Previous prevalence studies have often had restricted sample selection of stroke patients leading to sub-population analyses, and not controlling for age and gender in comparison populations [32–34]. The present study firstly aimed to determine the prevalence among a less selective stroke population three months after stroke, and to directly compare prevalence with an age and gender matched general population sample from a similar region. We hypothesized that the fatigue prevalence would be higher in the stroke sample. The second aim of this study was to investigate the relationship between motor activity early after stroke and PSF. Because some evidence exists supporting the positive impact of physical activity on fatigue, we hypothesized that patients engaged in more motor activity early after stroke would have reduced likelihood of fatigue at three months, after adjustment for stroke severity and pre-stroke function, and independent of depression, pain, pre-stroke fatigue, age and gender. A reversed causal pathway was also considered possible as the reduced activity early after stroke may be caused by fatigue which then persists three months after stroke [13].

## Methods

### Study design and settings

This was a prospective observational study including patients admitted to eleven Norwegian hospitals [35]. An age and gender matched control group was derived from a population-based study in the county of Nord-Trøndelag [36], were two of the eleven hospitals were located.

### Study participants

From 1st December 2011 to 11th June 2013, all consecutive acute first ever or recurrent stroke patients (except those with subarachnoid haemorrhages) admitted to the eleven stroke units were invited to participate, provided they were over 18 years of age, understood Norwegian and were not on palliative treatment. Stroke was defined according to the World Health Organisation definition. Recruitment was within 14 days after stroke onset. In keeping with Norwegian consent procedures, for patients unable to sign informed consent, verbal consent to participate was obtained from their next of kin. Further details of the study methods can be found in a prior publication [35]. Patients alive at three months, were contacted either in person or by telephone interview for assessment of perceptions of fatigue, depression, and pain.

Community-dwelling controls came from the Nord-Trøndelag population Health Survey3 (HUNT3-survey) [36]. The HUNT3-survey is a population-based study of the Norwegian county of Nord-Trøndelag. Two of the eleven hospitals in the stroke study were located in Nord-Trøndelag. Data were collected from October 2006 to June 2008. All adult residents aged ≥ 20 years were invited to participate in the study. The HUNT3-survey included several priority public health issues, and questionnaires included fatigue [37, 38], as outlined below. Of 93,860 eligible adults, 50,807 (54.1 %) returned the questionnaire and written consent. Participation was highest among people 60–69 years (71 %) decreasing to 18 % in the oldest age group 90–96 years. There was a selection bias toward more healthy individuals and higher socioeconomic status [39].

### Ethics

The Regional Committee for Medical and Health Research Ethics in Central Norway approved the study and storage of data on behalf of all participating hospitals and also the use of data from the HUNT3-survey (REC numbers 2011/1428 and 2012/675 respectively).

### Baseline assessment of stroke patients

Baseline characteristics of the stroke participants measured at inclusion included age, gender, pre-stroke function measured by modified Rankin scale (mRS) [40], stroke severity measured using National Institutes of Health Stroke Scale (NIHSS) [41], stroke type by Oxford classification [42], co-morbidities and pre-stroke fatigue. Pre-stroke fatigue was estimated from the following two items: ‘Did you experience fatigue before you had your stroke’ (yes/no), and, ‘If yes, how long did you experience fatigue’ (less than a week, less than three months, 3–6 months and more than six months). Patients who reported fatigue lasting longer than three months before the stroke were classified as having pre-stroke fatigue [11].

For the early motor activity outcomes, participants were observed every 10th minute during a working day from 8.00 am to 5.00 pm using the method of *behavioural mapping*. Motor activity was defined as the proportions of the daytime spent (i) *in bed*, (ii) *sitting out of bed* and (iii) *upright.* The procedure was reported in detail in a previous publication [43].

### Data extracted from the HUNT3-survey

Age and gender were used to select the general population sample from HUNT3-survey participants and data collected from the matched participants included comorbidities and fatigue.

### Outcome measures

Stroke patients were assessed three months after the stroke. Fatigue was measured in both samples (stroke and controls) using a simple fatigue questionnaire from the HUNT3-survey. This was a single question about weariness/fatigue: “Do you feel, for the most part, strong and fit or tired and worn out?”. There were seven response categories which ranged from “1 = very fit and healthy” to “7 = very tired and worn out”, with the middle option as neutral. Fatigue was defined as a score ≥ 5. In the stroke group, a second fatigue questionnaire, the Fatigue Severity Scale (FSS) was also administered. The 9-item FSS is the most commonly used scale to measure fatigue in stroke patients [16, 28, 44]. The shorter 7-item version of the FSS, FSS-7, was shown to have better psychometric properties in patients with stroke than the original 9-item version [2]. The FSS-7 was therefore chosen for this study.

Pain was assessed by a simple question ‘Did you experience new pain after stroke? (yes/no)’. Depression was assessed by Hospital Anxiety and Depression Scale (HADS) [45]. HADS is a self-report questionnaire which comprises two subscales HADS-anxiety and HADS-depression (HADS-D), each with seven items scored from zero to three. The scores are summed to give a total score for each subscale ranging from 0 to 21.

### Data management and analysis

Stroke patients were matched by age (up to a maximum of 2 years difference) and gender to respondents from the HUNT3-survey [36] who had all the outcome measures of interest to this study and no history of previous stroke. The HUNT participant of the same gender with the closest age (in 0.1 year increments) to each stroke participant was selected, with the matching procedure carried out blinded to any other outcome measure. The number of participants with available data determined the sample size for the study.

FSS scores from the 7-point Likert scale response options were averaged to yield a score from 1.0 to 7.0. Higher scores indicate higher fatigue levels. Most studies recommend a cut-off score of ≥ 4.0 as indicative of fatigue [33, 46]. The FSS-7 and HADS-D questionnaires were excluded if less than four items were answered. Up to three missing items were imputed with the average of the answered items.

Fatigue prevalence was examined in both groups using the HUNT3-survey questionnaire. The proportion of participants from each group reporting fatigue ≥ 5 on this questionnaire was compared using the chi-square test. Fatigue prevalence among the stroke patients was also reported using the FSS-7 with cut off of ≥ 4.0.

The association between early motor activity and PSF was tested using logistic regression models with fatigue dichotomised using FSS-7 score ≥ 4.0. Proportion of daytime *in bed*, *sitting out of bed* and *upright* were each tested in separate simple models. Stroke severity (NIHSS score) and pre-stroke function (mRS) were included as covariates. A single comprehensive multivariable logistic regression model also including HADS-D score, pain, pre-stroke fatigue, age and gender as additional independent variables was also examined. In this model both *time in bed* and *time upright* were included but *time sitting out of bed* was excluded as it is co-dependent on the other two activity categories. This model was designed to determine whether early motor activity or inactivity were independently associated with fatigue at 3 months.

## Results

**Fig. 1:**
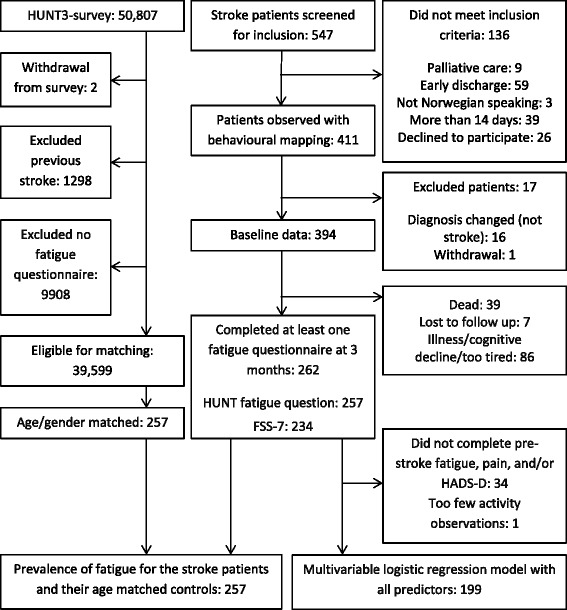
Flow of participants through the study

Two hundred and fifty-seven stroke participants were age and gender matched to HUNT3-survey participants for the prevalence study, and 199 stroke participants had the outcome measures needed for inclusion in the regression models (Fig. 1). Four patients had missing items on the FSS-7 questionnaire (one had three items missing and three had one item missing) and had the missing data imputed. There was a mean of 4.2 (SD 2.8) days from admission to the stroke unit to the day of inclusion in the study and behavioural mapping. Table 1 shows the descriptive data and fatigue prevalence for the age-gender-matched cohort. Data were available in both groups for several co-morbid diseases. These were hypertension, heart failure, myocardial infarct, lung disease (including asthma and COPD), kidney disease, diabetes mellitus, cancer and connective tissue disease (including rheumatoid arthritis and spondylitis). Thirty-four percent of the HUNT3-survey cohort had none of these diseases, while only 16 % of the stroke patients had none. Twenty-five percent of the stroke patients had three or more of the diseases, compared with only 11 % in the general population. Most patients were classified as PACI (40 %) according to the Oxford Classification, with only 7 % as TACI. Prevalence of fatigue ranged from 24 to 41 % across the different classification groups using the HUNT3 survey fatigue question, and ranged from 35 to 44 % using FSS-7.

**Table 1:** Descriptive data and prevalence

			Stroke	HUNT3-survey
Gender, % female			46.3 %	46.3 %
Age, mean (SD, range)			74.8 (11.4, 30.7–91.7)	74.8 (11.5, 30.7–92.5)
HUNT3 fatigue question (Do you feel, for the most part, strong and fit or tired and worn out?), n (%)	1. Very strong and fit		6 (2.3 %)	11 (4.3 %)
	2. Strong and fit		29 (11.3 %)	42 (16.3 %)
	3. Somewhat strong and fit		60 (23.3 %)	88 (34.2 %)
	4. Somewhat in between		82 (31.9 %)	88 (34.2 %)
	5. Somewhat tired and worn out		42 (16.3 %)	21 (8.2 %)
	6. Tired and worn out		20 (7.8 %)	6 (2.3 %)
	7. Very tired and worn out		18 (7.0 %)	1 (0.4 %)
Fatigue (HUNT3), % score ≥ 5			31.1 %	10.9 %
Fatigue (FSS-7), % score ≥ 4.0			34.6 %	-
Early motor activity, mean (SD)	% of day in bed		36.6 (23.4)	-
	% of day sitting out of bed		47.4 (19.9)	-
	% of day upright		10.9 (9.2)	-
	% of day not observed		5.1 (7.9)	-
Stroke severity (NIHSS), mean (SD)			5.0 (5.0)	
Function at inclusion (mRS), median, mean (SD)			3, 3.2 (1.1)	-
Function at 3 months (mRS), median, mean (SD)			2, 2.5 (1.2)	-
		n (% of cohort)	% reporting fatigue with HUNT3, FSS-7	
Oxford stroke classification groups	TACI	17 (7 %)	41 %, 38 %	-
	PACI	103 (40 %)	34 %, 40 %	-
	LACI	58 (23 %)	24 %, 35 %	-
	POCI	47 (18 %)	30 %, 44 %	-
	Haemorrhagic	32 (13 %)	31 %, 35 %	-
Co-morbidities, % of cohort	Hypertension		68 %	48 %
	Heart failure		10 %	7 %
	Myocardial infarct		19 %	14 %
	Lung disease (including asthma and COPD)		12 %	14 %
	Kidney disease		3 %	2 %
	Diabetes mellitus		15 %	11 %
	Cancer		18 %	9 %
	Connective tissue disease (including rheumatoid arthritis and spondylitis		8 %	8 %

Descriptive data, prevalence of fatigue and early motor activity data are provided for the stroke patients (*n* = 257) and their age/gender-matched counterparts (*n* = 257) from HUNT3-Survey

*FSS-7* 7-item Fatigue Severity Scale, *mRS* modified Rankin Scale (range of scores 0–5), *NIHSS* National Institutes of Health Stroke Scale (range of scores 0–42), *COPD* chronic obstructive pulmonary disease

Chi-square test indicated a significant difference in fatigue prevalence between the groups (31.1 % among stroke versus 10.9 % among healthy controls, *p* < 0.001). Odds of a stroke patient experiencing fatigue three months after stroke were 3.7 times the odds for the general population. The prevalence of fatigue was broadly similar if using the FSS-7 scale with a cut off of ≥ 4.0 for fatigue or using the HUNT3-survey fatigue question with cut off of ≥ 5 (34.6 and 31.1 % respectively).

The simple regression models testing the association of each of the early motor activity variables with fatigue (controlling for pre-stroke function and stroke severity) showed no association: proportion of *time in bed* OR 95 % CI 0.99–1.02 (*p* = 0.14), *time sitting out of bed* OR 95 % CI 0.98–1.01 (*p* = 0.21), and *time upright* OR 95 % CI 0.96–1.03 (*p* = 0.58). In the comprehensive model, which included the independent variables in the simple models plus age, gender, pre-stroke fatigue, depression, and pain, only pre-stroke fatigue, depression and pain were significantly associated with fatigue at three months (Table 2). Proportion of *time in bed* approached significance (*p* = 0.058). If the point estimate for *time in bed* of B = 0.02 was correct, then for every additional 1 % of the daytime (approximately 5.4 min) spent in bed, there was 2 % greater odds of experiencing fatigue at three months, holding all other variables constant.

**Table 2:** Descriptive data and results of comprehensive multiple variable regression model

Independent variables		B	OR (95 % CI)
Gender, n (%) female	91 (45.7 %)	−0.58	0.56 (0.26–1.21)
Age, mean (SD, range)	73.8 (11.7, 30.7–91.3)	−0.002	1.00 (0.97–1.03)
Pre-stroke fatigue, n (%) yes	53 (26.6 %)	1.30*	3.67 (1.62–8.31)
Depression (HADS-D), mean (SD)	3.8 (3.8)	0.27*	1.31 (1.17–1.47)
Pain (new since stroke), n (%) yes	38 (19.1 %)	1.51*	4.55 (1.82–11.34)
Pre-stroke function (mRS), mean (SD)	1.4 (1.1)	0.04	1.04 (0.70–1.54)
Stroke severity (NIHSS), mean (SD)	4.0 (3.7)	0.07	1.08 (0.97–1.19)
Early motor activity:			
% of day in bed, mean (SD)	35.0 (22.8)	0.02*	1.02 (1.00–1.04)
% of day upright, mean (SD)	11.8 (9.3)	0.03	1.03 (0.98–1.07)

*N* = 199, dependent variable fatigue (FSS-7 score ≥ 4.0), *significant at *p* < 0.05, *significant at *p* < 0.10 (trend). 77 participants (38.7 %) had fatigue

*HADS-D* Hospital Anxiety & Depression Scale – Depression subscale (range of scores 0–21), *mRS* modified Rankin Scale (range of scores 0–5), *NIHSS* National Institutes of Health Stroke Scale (range of scores 0–42)

Pre-stroke fatigue was one of the strongest independent predictors of PSF in our model (OR 3.7, 95 % CI 1.6–8.3, *p* = 0.002). The percentage of stroke patients that reported pre-stroke fatigue (had experienced fatigue prior to their stroke lasting at least three months) was 27 %, which was much higher than fatigue in the general population (11 %), although different measurement questionnaires were used. Of the 53 stroke participants reporting pre-stroke fatigue, 30 (57 %), reported fatigue at three months. However, about a third (32 %) of the 146 without pre-stroke fatigue reported fatigue three months after stroke.

## Discussion

The main finding from the study was, a higher prevalence of fatigue in stroke patients even after careful matching with a general population sample. Prevalence of fatigue three months after stroke was around one third, using either FSS-7 with a cut off ≥ 4.0, or using the HUNT3-survey questionnaire with a cut off ≥ 5. The prevalence is lower than most previous studies where prevalence was most often reported in the range of 50 %. There are several possible reasons for this difference. Most obviously, use of different questionnaires and different cut-offs to define fatigue will affect prevalence findings. However, a further possible explanation may be the older patient population in our study compared with other studies. Younger patients may be more aware of fatigue due to increased likelihood of wanting to return to work, more social activities, and higher activity levels [9, 12, 47]. We did not find compelling support for our hypothesis that more early motor activity would be associated with decreased likelihood of PSF. Our analysis confirms previous findings that pre-stoke fatigue, depression and pain are important predictors. *Time in bed* almost reached statistical significance in the model, with 95 % CI for OR ranging from 1.00 (no association) to 1.04 (4 % greater odds of having fatigue for every 5.4 min of extra bed rest).

The stroke patients were about three times more likely to report fatigue than their community-living counterparts who had not experienced stroke. Our results also showed that the stroke patients had more than double the likelihood of having at least one other disease prior to their stroke compared to the general population, and more than double the likelihood of having three or more other diseases. This finding suggests that the higher prevalence of PSF may be at least in part related to the stroke population being in poorer health even before they had a stroke. The previous literature on the association between pre-stroke co-morbidities and fatigue is not clear. A study in young patients found an association between PSF and both diabetes mellitus and myocardial infarction [32], while two other studies found no such association [14, 15]. PSF is a serious problem which clearly warrants better monitoring and management. Our findings suggest that pre-stroke health is an important factor in development of PSF.

Our findings hint at the possibility that early inactivity may be associated with fatigue at three months. This may be similar to the finding that more time in bed, but not less time in higher level activities, was predictive of worse functional outcome three months after stroke [48]. Previous bed rest studies have shown bed rest in general is not a benign treatment, but harmful to health [49, 50]. One possible mechanism by which bed rest could lead to higher levels of fatigue is the loss of cardiorespiratory fitness (CRF). CRF declines rapidly with bed rest [51], and is related to fatigue scores [52]. However, a recent review of cross-sectional studies found neither current physical activity levels nor CRF explained the level of fatigue experienced by people after stroke [53]. The risk of immobility-related complications increases with increased amounts of bed rest [54] suggesting that an association between fatigue and time in bed might also be explained by an increased prevalence of post-stroke complications. The reverse causal pathway is also plausible, whereby early activity is dependent on the absence of fatigue. Despite our non-significant finding, we argue that further research is still needed to investigate how early fatigue and early inactivity are related to the problem of debilitating PSF.

A recent study found that patients with stroke, who had more effortful movement as determined by movement velocity during a timed hand movement task, were found to have increased likelihood of fatigue [22]. The authors proposed that the relationship could be due to either a simple effort-fatigue relationship or because both fatigue and reduced movement speed may result from an alteration in motor cortex excitability. With this finding in mind alongside our own results, PSF may be largely explained by a combination of poor pre-stroke health, effects of the brain injury (including early inflammatory effects), issues secondary to stroke during the acute phase (including medications, sleep problems and complications), depression, pain, the harmful consequences of too much inactivity, and increased effort of movement related to motor impairment.

Strengths of our study of PSF prevalence are the largely unselected stroke sample and the appropriate and well-matched control group. The main limitations of our study are that important confounding variables may be missing from the regression models such as cognitive function, medications and sleep disorders [19]. However, all models were adjusted for the most common and significant predicting variables after stroke. Secondly, there may be bias introduced because participants excluded due to lost to follow-up (*n* = 7), illness/cognitive decline/too tired (*n* = 86), or failure to complete pre-stroke fatigue, pain or depression questionnaires (*n* = 34) was potentially non-random. This group was likely to include the least healthy among the cohort. Thirdly, our measures of activity early after stroke may not adequately represent activity, or inactivity, of importance in preventing the development of PSF. All studies of PSF are limited by the multidimensional nature of fatigue and the inadequacies of the fatigue measurement tools used. Pre-stroke fatigue was measured with a different questionnaire to PSF, which may compromise our study, and early PSF was not measured. Finally, as the stroke units were all in Norway where national guidelines strongly recommend promotion of early out of bed activity, there may not have been sufficient between-individual spread of inactivity/activity levels for the role of early motor activity in predicting PSF to be revealed. The likelihood that the amount of bedrest is closely related to stroke severity and pre-stroke function also poses a challenge in this and future studies. Carefully controlling for these confounders as in the present study, using pre-stroke mRS and NIHSS in the models, is helpful but may still be inadequate. These limitations may have resulted in the lack of support for our second hypothesis.

Some previous research supports there being a difference between mental and physical fatigue, particularly after stroke. The impact of a stroke (irrespective of whether ischemic or haemorrhagic) taxes the central nervous system and increases the level of cognitive strain, which may be interpreted as fatigue. Stroke patients may be physically capable of participating in rehabilitation exercises or physical activity, but feel unable to engage in the activity due a depletion of cognitive reserves or higher vascular burden. Global increases in allostatic load coupled with negative affect may further compound this problem. Drawing a distinction between mental and physical fatigue is currently difficult and controversial and was not attempted in our study. However, we suggest further research along these lines may yield important knowledge and facilitate management of the problem of PSF in the future.

PSF presents management challenges with few options currently available with proven effectiveness [55]. A combined cognitive therapy and graded exercise program has shown promise in alleviating fatigue, as well as cognitive therapy alone [56, 57]. However these trials are small and more research is needed on the effect of multifactorial approaches including exercise programs. It is apparent from the results of observational studies that improvement of general health and management of depression, sleep and pain should all help alleviate PSF. We also suggest that determining the appropriate amount of time spent on bed-rest versus out of bed activities early after stroke warrants urgent further investigation in relation to fatigue [31].

## Conclusions

Despite the lower prevalence of PSF in this relatively unselected stroke population than typically previously reported, this study confirms a higher prevalence than in those without stroke and further highlights the problem of PSF. Pre-stroke health appears to be an important factor, as does post-stroke depression and pain. The role of early motor activity in the development of fatigue following stroke remains unclear.

## Abbreviations

NIHSS: National Institutes of Health Stroke Scale
mRS: Modified Rankin Scale
FSS-7: Seven-item Fatigue Severity Scale
HADS-D: Hospital Anxiety & Depression Scale – Depression subscale
OR: Odds ratio
CI: Confidence interval
